# Semi-Feral Horse Grazing Benefits the Grassland Diversity of Flowering Plants Including a Pollinator-Promoting Indicator Species

**DOI:** 10.3390/ani15060862

**Published:** 2025-03-17

**Authors:** Carl-Gustaf Thulin, Yufei Chen, Pablo Garrido

**Affiliations:** 1 Department of Animal Biosciences, Swedish University of Agricultural Sciences, 750 07 Uppsala, Sweden; yien0002@stud.slu.se (Y.C.); pablo.garrido@patrimonionatural.org (P.G.); 2 Natural Capital Foundation (Fundación Patrimonio Natural de Castilla y León), 470 08 Valladolid, Spain

**Keywords:** biodiversity, diversity index, *Equus*, grazing impact, restoration, rewilding

## Abstract

European grasslands and their biodiversity are declining rapidly due to land use changes, which highlight the need to develop effective restoration strategies. This study investigates the impact of reintroducing the Swedish national horse breed (the Gotland Russ) on grassland plant diversity and evenness in abandoned agricultural landscapes in Southeast Sweden. Twelve horses were introduced into three 10–13-hectare enclosure replicates (four horses per enclosure) in a three-year (2014–2016) rewilding experiment. Plant species richness, evenness, and diversity were investigated in both grazed and un-grazed conditions. The results indicate that horse grazing significantly increased grassland plant species diversity and richness, with higher Shannon and Simpson’s diversity indices in grazed areas. In addition, the abundance of white clover (*Trifolium repens*), a signal species beneficial to pollinators, increased significantly in grazed areas. These findings emphasize the need for integrating large herbivore grazing into ecological restoration practices. Considering the recently enacted EU Nature Restoration Law, which aims to restore 20% of Europe’s degraded ecosystems by 2030, this research provides critical insights into scalable restoration methods. The implementation of restoration strategies that include large herbivores may enhance the resilience and biodiversity of European grasslands, thereby aligning with the EU’s restoration goals.

## Simple Summary

Wild horses once roamed large areas of Eurasia but were extinct by the end of the 19th century. Meanwhile, domestic horses dwell in large numbers. Domestic horses are, however, unable to fulfil the ecological functions of wild horses due in part to their management. Here, we investigate whether wilder, or semi-feral, horse management with year-round grazing and no supplementary feeding may benefit floral diversity. In an experiment with the Swedish national horse breed, the Gotland Russ, we show that the floral diversity increases with grazing, while an indicator species of clover, which is beneficial to pollinators, also benefits. This suggests that horses could be used for ecosystem restoration purposes as part of the obligations taken by EU member states under the new European Nature Restoration Law.

## 1. Introduction

The late Quaternary loss of large herbivores (>45 kg) led to a drastic reduction in herbivory functional diversity, with significant effects on ecosystems [1,2,3,4]. Large wild herbivores were a fundamental component of European landscapes, playing key roles in ecosystem functioning and maintaining open landscape structures [5]. The defaunation processes produced significant ecological state shifts in different biomes [6] with cascading effects on plant community composition, vegetation structure, fire regimes [7], and even climate [8,9].

The current consensus is that large and megaherbivores were largely decimated as a consequence of modern human expansion [10,11,12]. Recent estimates showed a global reduction in megafauna diversity in the Late Pleistocene greater than 50% [12]. Europe’s megafauna species richness has declined by around 71% and its biomass by nearly 95% [3]. The European wild horse, the tarpan (*Equus ferus ferus*), was extinct at the turn of the last century [13] and was thus lost as a component of the pan-European landscape. Horses were domesticated around 5500 years before the present, initially for warfare, transport, and agriculture [14], but during the 20th century, they were increasingly used for competition, pleasure, and leisure [15]. Even though domesticated horses may, to some degree, fulfil the ecosystem functions of wild ungulates [16,17], most horses are today kept in such a way that their impact on the surrounding landscape is limited [18,19].

Horses are functional grazers, influencing plant community dynamics through foraging and trampling [19], as natural fertilizers [20] and plant dispersers [21]. Wild horses may also benefit plant species richness, evenness, and heterogeneity more than domestic ruminants do [19,22]. Restoring extinct large wild herbivore communities may, therefore, benefit biodiversity and ecosystem function [4,23,24].

Restoration practices using extinct species or extant functional proxies for ecosystem restoration, i.e., trophic rewilding [25], is gaining momentum in Europe as a restoration practice. Yet, empirical rewilding experiments are still limited. Here, we test how the reintroduction of a functional substitute of a wild horse affects the plant diversity and species richness of flowering plants. In addition, we investigate the impact of horse grazing on *Trifolium* sp., a signal species beneficial to pollinators.

## 2. Materials and Methods

### 2.1. Experimental Design

**Figure 1:**
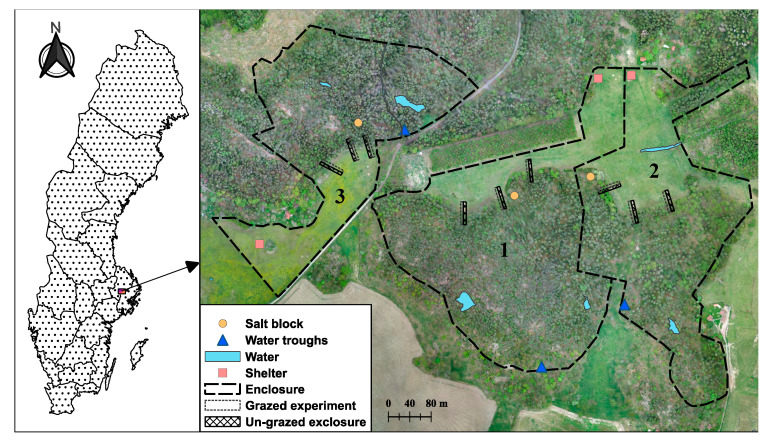
Location and experimental design at Krusenberg estate in Southeastern Sweden with experimental areas 1, 2, and 3 highlighted [27].

To mimic wild horse ecosystem function, twelve one-year-old Gotland Russ horses (*Equus ferus caballus*), the Swedish national horse breed, were kept year-round without supplementary feeding in three enclosure replicates (sized ca. 10–13 hectares) between May 2014 and September 2016 on Krusenberg estate, a property of the Swedish University of Agricultural Sciences (SLU) near Uppsala, Sweden (59°44′8″ N, 17°38′58″ E) (Figure 1) [23,26]. The enclosures were composed of approximately one-third grassland and two-thirds mostly mature forest [23,26]. In each enclosure, three rectangular exclosures (size 42.5 × 5 m, located 20 m into the forest and 22.5 m into the grassland) were set up, i.e., there were a total of nine. The exclosures were centered over the edge zone between the forest and grassland, which in general is a biologically rich area (Figure 1). An experimental (grazed) area of equal size was delineated parallel to each exclosure (Figure 1). Exclosures were fenced to prevent horses from grazing and, thus, mimicked land abandonment. Prior to the experiment, the experimental area was mowed occasionally and sometimes grazed with cattle [23].

The Gotland Russ were yearling stallions with an average body weight of 185 ± 21 kg and in appropriate body condition at the start of the study. They were acclimatized in an adjacent enclosure at the experimental location for one month before the experiment started, then allocated into groups of four individuals in each experimental enclosure [26]. Grazing pressure was 0.35 horse/hectare, with an average adult horse body mass of 250 kg (i.e., approximately 90 kg horse/hectare) [23]. Each enclosure was expected to meet the energy requirements of the horses based on prior estimates of grassland productivity [26]. The experiment used a crossover design, in which horse groups were alternated between the three enclosures each spring (right before plant production season started), so all groups spent one plant production season in each enclosure. Each enclosure included a 16 m^2^ shelter, water *ad libitum*, and a salt block with trace minerals. A total of four horses were temporarily removed from the experiment due to low body condition during the winter of 2014, and one individual was excluded from the study due to injury in January 2016 and was not replaced [28]. Thus, during the final six months of the experiment, the overall grazing pressure was lowered with one horse (out of twelve).The experimental design was approved by the Uppsala Animal Welfare Ethics Committee (protocol C28/14, 28 April 2014).

**Figure 2:**
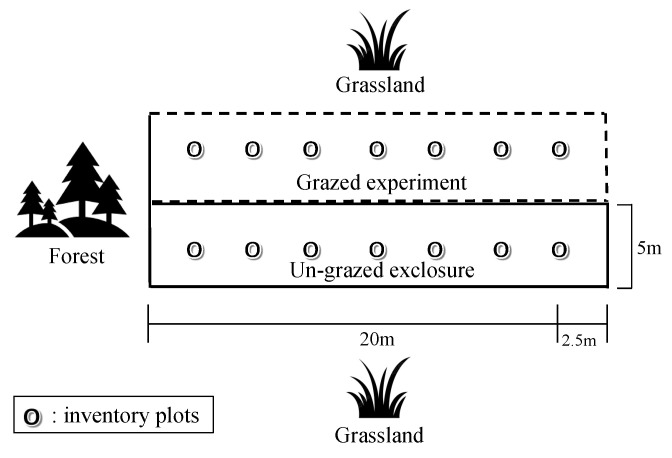
Vegetation survey design. Seven paired grazed and un-grazed inventory plots on grasslands were surveyed (black circles indicate the inventory plots) [27].

Surveys of flowering plants were conducted in seven paired permanent inventory plots on the grassland section of each exclosure, paralleled with equal plots in the grazed area. All seven plots were 0.25 m^2^ each (0.282 m radius) spaced 2.5 m apart (Figure 2), marked with black plastic needles hammered into the soil in the plot center. For each plot pair (grazed–ungrazed), all plants were identified at species level and their abundance (in percentage of plot cover) assessed visually using a quadrat as permanent reference frame. Percentage accuracy was 1% steps up until 10% and thereafter in 5% intervals. Vegetation surveys were performed in July and September in 2014, as well as in May, July, and September during the two subsequent years 2015 and 2016 [23]. Plants within the plots were visually identified at species level and their abundance was noted as percentage (%). To enable comparable analysis over three years of experimental grazing during the plant growth peak season in mid-summer, only data from July each year were utilized for this study. Vegetation surveys were performed by two different observers, one in 2014, and the other in 2015 and 2016.

### 2.2. Data Analysis

First, differences in plant species diversity and evenness between treatments (grazed and un-grazed) and time were calculated as Shannon–Winner diversity index, Simpson’s diversity index, and Pielou’s evenness index using the diversity function in package “vegan” [29].

Shannon–Winner diversity index (*H*) was calculated as follows:

$$\left(H\right)=-\sum_{i-1}^{S}p_{i}lnp_{i}$$

where

*S* = total number of species (species richness);
*p_i_* = proportion of individuals belonging to species *i* (calculated as proportion of species *i* = *n_i_/N*, where *n_i_* is the number of individuals of species *i* and *N* is the total number of individuals of all species);
ln = natural logarithm.

Simpson’s diversity index (*D*) was calculated as follows:

(D)=∑i=1Sni−N2

where

*S* = total number of species (species richness);

*n_i_* = number of individuals of species *i*;

*N* = total number of individuals of all species.

Pielou’s evenness index (*D_pie_*) was calculated as follows:

$$D_{pie}=\frac{\left(H\right)}{lnS}$$

where

(*H*) = Shannon–Winner diversity index;

*S* = total number of species (species richness);

*ln* = natural logarithm.

Wilcoxon signed-rank test was used to assess whether there were any significant differences among grazed and un-grazed conditions through time within each of the calculated biodiversity indices (differences were considered significant at *p* < 0.05).

Second, we tested how plant species richness was affected by horse grazing and time by fitting a generalized linear mixed-effects model (GLMM) with Poisson distribution (log-link), including an interaction effect between treatment and time as well as a random effect for plots within enclosures and exclosures using the function glmer in package “lme4” [30]. Plant species richness was calculated as the number of plant species per plot.

Third, the grazing impact on the abundance of three *Trifolium* species, *T. medium* (zigzag clover), *T. pratense* (red clover), and *T. repens* (white clover), used as indicator species [31] for durability (*T. medium*), plasticity (*T. pratense*), and adaptive capacity and pollinator attractiveness (*T. repens*) [32,33,34,35], was assessed to fit a GLMM with Poisson distribution (log-link) using package “lme4” [30], including an interaction effect between treatments and time as predictor. These models also included a random effect term (plots within exclosures and enclosures, respectively). The results for *T. medium* and *T. pratense* were excluded from analyses due to low number of observations. All statistical analyses were performed and plots were made using R program version 4.3.2 [36].

## 3. Results

**Figure 3:**
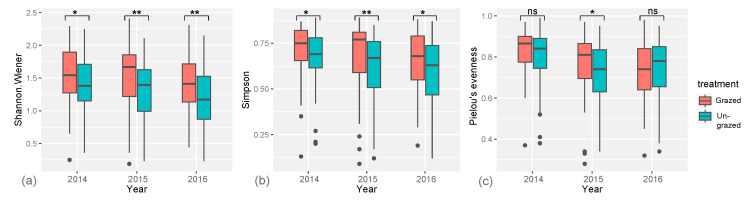
Box-plots representing the following: (**a**) The Shannon–Winner diversity index; (**b**) Simpson’s diversity index; and (**c**) Pielou’s evenness index for comparisons between treatments (grazed vs. un-grazed) within the three years of the experiment [36]. The horizontal line inside boxes represents the mean value, while black dots note outliers. Significant symbol: *p* ≤ 0.01: “**”; *p* ≤ 0.05: “*”; *p* > 0.05: “non-significant”.

A total of 94 different species of flowering plants were recorded in July during the three-year experiment (see Supplementary Material). The Shannon diversity index was significantly higher in the grazed (2014: *p* = 0.028; 2015 and 2016: *p* < 0.01) compared to the un-grazed conditions (Figure 3a). The Simpson’s diversity index also corroborated that the grassland plant diversity was higher in the grazed compared to the un-grazed areas (2014: *p* = 0.04; 2015: *p* < 0.01; 2016: *p* = 0.034) (Figure 3b). The plant species evenness was higher in the grazed conditions in 2015 (*p* = 0.02), but we did not find any significant differences in 2014 and 2016 (Figure 3c).

In general, the plant species richness was higher (although not significant) in the grazed compared to the un-grazed areas (see Table 1). Moreover, the plant species richness significantly increased with time and treatment, i.e., grazing (Table 1). The abundance of the indicator species *T. repens* was higher in the grazed conditions (Table 2), while it declined over time in the un-grazed areas (*p* < 0.001; Table 2).

## 4. Discussion

This study shows that domestic horses can benefit floral diversity and support indicator species, which may have positive effects on pollinator communities [23] and therefore restore the ecological functions of extinct wild horses in grassland and wood-pasture ecosystems. The Shannon and Simpson’s diversity indexes were higher in the grazed compared to the un-grazed conditions, which suggests that grazing had a positive effect on the overall diversity of flowering herbaceous plants. In addition, the Shannon diversity index, which reflects both richness and evenness, was significantly higher in grazed areas, indicating a more even distribution of species abundances. Similar results were shown by Marion et al. (2010) and Li et al. (2021) [37,38], who found that grazing by horses, cattle, and sheep contributed to increases in Shannon diversity scores in France and China, respectively. Its noteworthy that our results were obtained despite that a total of four horses were temporarily removed from the experiment due to low body condition during the winter of 2014, and one individual was excluded from the study due to injury in January 2016 and not replaced, thus temporarily lowering the overall grazing pressure.

The higher values of Simpson’s diversity index suggest a reduction in competitive species, with a more equitable distribution of species abundances induced by grazing, which resulted in increased plant diversity [23]. Plant evenness, however, was only significantly higher in the grazed conditions in 2015, which may suggest that grazing induced a redistribution of plant species abundances in the grassland (see Figure 3c).

The plant species richness was also higher in the grazed compared to ungrazed conditions (Table 1). Positive effects of grazing on plant species richness have also been shown for sheep grazing [39,40], semi-feral horses and cattle grazing [41], horse grazing [19,22,42], and cattle grazing [43]. One of the pathways in which herbivore grazing may have positive effects on plant diversity and richness is by limiting light competition [44] and increasing sunlight availability for short plants and seeds to grow [45]. Moreover, large herbivores including horses may also exert ecosystem engineering effects through forest compositional and structural changes [18], enhancing water availability [46], and creating different microhabitats such as defecation concentrations (latrines) [47] and wallowing areas that support higher arthropod diversity [48,49,50]. The grazing and browsing preferences of horses may also result in positive impacts on pasture diversity, increased forb cover [23,43], and pollinator diversity [22].

Indicator species can be used to determine grassland plant diversity or environmental conditions, using only a small number of plant species [51]. In this study, *T. repens* increased in the grazed areas but declined in the ungrazed conditions. These differential responses highlight the importance of considering individual species’ dynamics when assessing the ecological impacts of grazing [52]. Grazing can affect species composition, distribution, and abundance, potentially altering competitive interactions and resource availability within the plant community [53,54]. *Trifolium* species are all perennial, while *T. repens* is a competitive species, which adapts to harsh climates and soil conditions better than other species of clover [34]. Recent research suggested that an increase in *T. repens* abundance favored the abundance and diversity of wild bees [35], and *Trifolium* species in general are important food resources for pollinating insects [55]. Thus, the increase in *T. repens* abundance in this study might explain a higher level of butterfly and bumblebee richness [23].

In Sweden, where this experiment was conducted, the primary threats to biodiversity in agricultural ecosystems are attributed to the intensification and abandonment of traditional agricultural practices [56]. During the mid-20th century, agricultural abandonment, reduced semi-natural pastures and meadows, and shrubification threatened the rural landscape biodiversity [57]. Notably, approximately half of Sweden’s red-listed species rely on farmed landscapes for their survival [58]. Here, we show that reintroducing an ecologically functional substitute of extinct wild horses could tackle current biodiversity declines and restore threatened grassland ecosystems. Although a consensus is still missing, all alternatives to revert the detrimental effects of land abandonment should be advocated to reduce current biodiversity declines [59]. Thus, a proportion of the 355,500 horses in Sweden [60], primarily kept for competition and leisure, could fulfil important ecosystem services and functions and partly alleviate the urgent need for nature restoration to tackle current multi-level biodiversity and climate crises.

## 5. Conclusions

We show that floral diversity was higher in experimental than control areas, and that plant species richness increased with time and treatment (Table 1) in our experimental conditions (0.35 horse/ha) where horses were kept year around without supplementary feeding and with a uniform stocking density over time. Our results indicate that horse grazing may be an important avenue for natural grassland and wood-pasture restoration, with positive effects on plant species diversity and richness. Given that the newly adopted EU Nature Restoration Law [61] urges EU member states to restore at least 20% of EU degraded natural habitats by 2030, urgent actions to tackle current biodiversity declines and to provide natural climate solutions and services [62] should be implemented without delay, especially in agricultural landscapes.

## Figures and Tables

**Table 1:** Results regarding the impact of experimental treatment (grazed and un-grazed) and time on plant species richness fitted to a GLMM. β = model regression coefficient estimate. SE = standard error. N = 378.

	GLMM Plant Species Richness			
Main Effects	β	SE	z Value	*p*-Value
Intercept	1.8	0.11	16.4	<0.001
2015	0.24	0.07	3.4	<0.001
2016	0.16	0.69	2.27	<0.05
Un-grazed	−0.12	0.07	−1.67	0.1
Interaction effects				
2015: un-grazed	−0.05	0.09	−0.52	0.6
2016: un-grazed	−0.23	0.1	−2.24	<0.05

**Table 2:** Results regarding the impact of experimental treatment (grazed and un-grazed) and time on plant abundance of *Trifolium repens* fitted to a GLMM. β = model regression coefficient estimate. SE = standard error. N =216.

	GLMM Plant Abundance of *Trifolium repens*			
Main Effects	β	SE	z Value	*p*-Value
Intercept	2.2	0.12	18.6	<0.001
2015	0.15	0.06	2.4	<0.05
2016	0.44	0.06	7.32	<0.001
Un-grazed	−0.4	0.08	−5.12	<0.001
Interaction effects				
2015: un-grazed	−1.63	0.18	−9.35	<0.001
2016: un-grazed	−2.02	0.38	−5.21	<0.001

## Data Availability

The dataset analyzed in this study is available as Supplementary Materials in a formal deposit or directly from the corresponding author on request.

## Supplementary Materials

The following supporting information can be downloaded at: https://www.mdpi.com/article/10.3390/ani15060862/s1, A list of the 94 species of flowering plants that were recorded in July during the three-year experiment is provided in Supplementary Materials.
