# Supplementary material for: Semi-Feral Horse Grazing Benefits the Grassland Diversity of Flowering Plants Including a Pollinator-Promoting Indicator Species
Source: Animals (Basel). 2025 Mar 17;15(6):862. doi: 10.3390/ani15060862 (PMC11939236; doi:10.3390/ani15060862)
Supplement: Supplementary file 1 [file animals-15-00862-s001.zip › animals-3515259-supplementary.pdf]

N	Species Name	Functional Type	Life Cycle
1	Achillea millefolium	Forb	Perennial
2	Aegopodium podagraria	Forb	Perennial
3	Alchemilla monticola	Forb	Perennial
4	Alchemilla sp	Forb	Perennial
5	Anchusa arvensis	Forb	Annual
6	Anemone nemorosa	Forb	Perennial
7	Anthriscus sylvestris	Forb	Perennial
8	Campanula rotundifolia	Forb	Perennial
9	Campanula sp	Forb	Perennial
10	Carex flava	Graminoid	Perennial
11	Carex leporina	Graminoid	Perennial
12	Carex pallescens	Graminoid	Perennial
13	Carex sp	Graminoid	Perennial
14	Centaurea jacea	Forb	Perennial
15	Centaurea montana	Forb	Perennial
16	Cerastium arvense	Forb	Perennial
17	Cerastium fontanum	Forb	Perennial
18	Cirsium arvense	Forb	Perennial
19	Cirsium palustre	Forb	Biennial/Perennial
20	Cirsium vulgare	Forb	Biennial
21	Cynosurus cristatus	Graminoid	Perennial
22	Epilobium sp	Forb	Annual/Perennial

23	Equisetum arvense	Fern	Perennial
24	Equisetum palustre	Fern	Perennial
25	Equisetum pratense	Fern	Perennial
26	Equisetum sylvaticum	Fern	Perennial
27	Festuca rubre	Graminoid	Perennial
28	Filipendula ulmaria	Forb	Perennial
29	Fragaria vesca	Forb	Perennial
30	Galium album	Forb	Perennial
31	Galium palustre	Forb	Perennial
32	Galium uliginosum	Forb	Perennial
33	Galium verum	Forb	Perennial
34	Geranium pratense	Forb	Perennial
35	Geranium sylvaticum	Forb	Perennial
36	Geum rivale	Forb	Perennial
37	Gnaphalium sylvaticum	Forb	Perennial
38	Hypericum maculatum	Forb	Perennial
39	Hypericum perforatum	Forb	Perennial
40	Juncus conglomeratus	Graminoid	Perennial
41	Juncus effusus	Graminoid	Perennial
42	Lathyrus palustris	Forb	Perennial
43	Lathyrus pratensis	Forb	Perennial
44	Leucanthemum vulgare	Forb	Perennial
45	Lotus corniculatus	Forb	Perennial

46	Luzula multiflora	Graminoid	Perennial
47	Luzula pilosa	Graminoid	Perennial
48	Melampyrum nemorosum	Forb	Annual
49	Melampyrum pratense	Forb	Annual
50	Melampyrum sylvaticum	Forb	Annual
51	Mentha arvensis	Forb	Perennial
52	Myosotis arvensis	Forb	Biennial/Perennial
53	Pilosella lactucella	Forb,Perennial	Perennial
54	Plantago major	Forb	Perennial
55	Poa trivialis	Graminoid	Perennial
56	Potentilla anserina	Forb	Perennial
57	Potentilla sp	Forb	Annual/Biennial/Perennial
58	Primula veris	Forb	Perennial
59	Prunella vulgaris	Forb	Perennial
60	Ranunculus acris	Forb	Perennial
61	Ranunculus auricomus	Forb	Perennial
62	Ranunculus repens	Forb	Perennial
63	Rhinanthus minor	Forb	Annual
64	Rhinanthus serotinus	Forb	Annual
65	Rumex acetosa	Forb	Perennial
66	Rumex acetosella	Forb	Perennial
67	Rumex crispus	Forb	Perennial
68	Rumex longifolius	Forb	Perennial

69	Rumex sp	Forb	Annual/Biennial/Perennial
70	Sagina procumbens	Forb	Perennial
71	Scorzoneroides autumnalis	Forb	Perennial
72	Stellaria graminea	Forb	Perennial
73	Stellaria longifolia	Forb	Perennial
74	Stellaria media	Forb	Annual
75	Succisa pratensis	Forb	Perennial
76	Taraxacum pallidipes	Forb	Perennial
77	Taraxacum vulgare	Forb	Perennial
78	Tragopogon pratensis	Forb	Biennial
79	Trifolium hybridum	Forb	Perennial
80	Trifolium medium	Forb	Perennial
81	Trifolium pratense	Forb	Perennial
82	Trifolium repens	Forb	Perennial
83	Tripleurospermum maritimum	Forb	Annual/Biennial/Perennial
84	Tripleurospermum perforatum	Forb	Annual
85	Tussilago farfara	Forb	Perennial
86	Urtica dioica	Forb	Perennial
87	Veronica arvensis	Forb	Annual
88	Veronica chamaedrys	Forb	Perennial
89	Veronica officinalis	Forb	Perennial
90	Veronica serpyllifolia	Forb	Perennial
91	Vicia cracca	Forb	Perennial

92	Vicia sepium	Forb	Perennial
93	Viola arvensis	Forb	Perennial
94	Viola riviniana	Forb	Perennial
